# Energy-Related Indicators and Breast Cancer Risk among White and Black Women

**DOI:** 10.1371/journal.pone.0125058

**Published:** 2015-04-30

**Authors:** Maureen Sanderson, Loren Lipworth, David Shen-Miller, Sarah Nechuta, Alicia Beeghly-Fadiel, Martha J. Shrubsole, Wei Zheng

**Affiliations:** 1 Department of Family and Community Medicine, Meharry Medical College, Nashville, TN, United States of America; 2 Division of Epidemiology, Department of Medicine, Vanderbilt Epidemiology Center, Vanderbilt-Ingram Cancer Center, Vanderbilt University School of Medicine, Nashville, TN, United States of America; 3 Department of Psychology, Tennessee State University, Nashville, TN, United States of America; Sudbury Regional Hospital, CANADA

## Abstract

Energy-related indicators, including physical activity, energy intake, body mass index (BMI) and adult weight change, have been linked to breast cancer risk. Very few studies of these associations have been conducted among black women, therefore we used the Nashville Breast Health Study (NBHS) to determine whether similar effects were seen in black and white women. The NBHS is a population-based case-control study of breast cancer among women age 25 to 75 years conducted between 2001 and 2010 in and around the Nashville Metropolitan area. Telephone interviews and self-administered food frequency questionnaires were completed with 2,614 incident breast cancer cases ascertained through hospitals and the statewide cancer registry, and 2,306 controls selected using random digit dialing. Among premenopausal white and black women, there was little effect of adult exercise or other energy-related indicators on breast cancer risk, regardless of tumor estrogen receptor (ER) status. The beneficial effect of adult exercise on postmenopausal breast cancer appeared to be comparable between white and black women (highest tertile relative to none - white odds ratio [OR] 0.8, 95% confidence interval [CI] 0.6-1.0, p for trend=0.05; black OR 0.7, 95% CI 0.4-1.1, p for trend=0.07); however, among black women the reduction was limited to those with ER-positive disease. White and black women should be encouraged to engage in more physical activity to reduce their risk of postmenopausal breast cancer.

## Introduction

Energy-related indicators, including physical activity, energy intake, body mass index (BMI) and adult weight change, have been linked to breast cancer risk. Increased physical activity has been associated with reduced breast cancer [1–7], which may be more pronounced for postmenopausal women [5–6, 8–9], for women who are lean/normal weight [1–2, 5] and for those who had lost weight during adulthood [5]. In contrast, we have reported higher risk of postmenopausal breast cancer in Chinese women who did not engage in physical activity and were either overweight or had gained a substantial amount of weight during adulthood [4]. Results of studies of energy intake and breast cancer, whether diagnosed premenopausally or postmenopausally [4, 10–12], have been inconclusive. The risk of breast cancer associated with high BMI differs by menopausal status, with a generally lower risk among premenopausal women [13–18] and a higher risk among postmenopausal women [13, 15–16, 19–21]. Similarly, breast cancer risk associated with adult weight gain also differs by menopausal status, with no increased risk among premenopausal women [22] and a higher risk among postmenopausal women [4, 22–23].

Aside from studies of BMI [14, 18–19, 24–30], very few investigations of these associations have been conducted among black women [7, 27–28, 31–35], therefore we used the Nashville Breast Health Study (NBHS) to examine whether similar associations were seen in black and white women.

## Materials and Methods

Detailed methods of this population-based case-control study conducted in and around the Nashville Metropolitan area appear elsewhere [36]. Briefly, cases aged 25–75 years, diagnosed with primary ductal carcinoma in situ or invasive breast cancer between February 2001 and December 2011, were identified through a rapid case ascertainment system utilized by five hospitals and the statewide cancer registry (n = 2,614, response rate 58%). Controls of similar age (5-year age groups), race, and county of residence were randomly selected utilizing random digit dialing (n = 2,306, response rate 48%). While the number of white cases and controls was approximately equal, there were approximately twice as many black cases as controls by design to enable future examination of genetic variants in this group. Written informed consent was obtained from subjects. The Institutional Review Boards of Vanderbilt University Medical Center, Meharry Medical College, St. Thomas Hospital, Centennial Hospital, and the Tennessee Department of Health approved this study’s protocol.

Trained interviewers conducted telephone interviews to ascertain information on demographic characteristics, suspected breast cancer risk factors, and energy-related indicators including adult exercise, occupational activity, current weight and height, and weight at age 18. Food frequency questionnaires validated in a similar Southern U.S. population [37] were administered to obtain information on another energy-related indicator, current diet. Information on exposures pertained to a period before an assigned reference date, the month and year of diagnosis for the cases and a similar date for controls. Adult exercise was calculated from the question “Over the past 10 years before (reference date), did you regularly engage in exercise activities? By regular, I mean totaling at least two hours per week continuously for at least six months in a row. Exclude activities which were part of your job or house work.” in which subjects could list the number of years, months per year and average hours per week for up to 5 activities. All reported exercises were converted to metabolic equivalent (MET) hours per day and summed [38]. Occupational activity was the sum from the question “In the past five years, during the time you were at work, how many hours per day, or per week were you standing or walking?” There was no measure of household physical activity. Total energy intake was calculated from the food frequency questionnaire as calories per day. Body mass index (BMI, kg/m^2^) was based on self-reported current height and weight one year prior to the interview. Weight change since age 18 was the difference in weight one year prior to the interview and self-reported weight at age 18 or about the time the subject may have graduated from high school. Adult exercise, occupational activity and weight change since age 18 were categorized as none (none or weight loss for weight change), and the remaining group of women were divided using tertiles among the controls for the main effects. Energy intake was categorized as quartiles among the controls for main effects. BMI was categorized using standard definitions of underweight/normal weight (<25), overweight (25–29.9), obese (30.0–34.9) and severely obese (≥35) for main effects. The referent group for the main effects was the lowest category for all energy-related indicators which for adult exercise was 0 MET hours per day reflecting the highest percentage of subjects. For most of the joint effects of energy-related indicators, the lower two of the four categories were collapsed; however, for adult exercise the upper two of the four categories were collapsed. The referent group for the joint effects was the highest category of adult exercise combined with the lowest category of energy intake or BMI or weight change. We also examined the main effects by tumor estrogen receptor (ER) status, available from pathology records for 70% of cases, using the same reference groups as the main effects, but collapsing the categories to form tertiles rather than quartiles.

Differences between cases and controls for demographic characteristics and breast cancer risk factors were assessed using t-tests and chi-square tests. Analyses are presented separately by race and menopausal status since the hypothesized effects of energy-related indicators on breast cancer may differ by these factors. Unconditional or polynomial logistic regression was used to estimate the odds ratio of breast cancer associated with the main and joint effects of energy-related indicators while controlling for potential confounding factors [39]. Interaction terms, the product of adult exercise and putative effect modifiers (energy intake, BMI and weight change), were added to logistic regression models and likelihood ratio tests were performed to test for effect modification. In addition to the other energy-related indicators, age, educational level, income, family history of breast cancer, history of benign breast disease, parity, age at first live birth, age at menarche, alcohol intake (drinking at least five alcoholic beverages a week for 12 months), cigarette smoking, use of oral contraceptives, use of hormone replacement therapy, and age at menopause as categorized in Table 1 were evaluated as potential confounders. Variables were considered confounders if their addition to the model changed the unadjusted odds ratio by 10 percent or more [40]. Tests for trend across categories of energy-related indicators were performed by entering categorical variables as continuous variables in the model. Statistical analyses were completed in SAS version 9.2 (SAS Institute, Cary, NC).

**Table 1:** Comparison of cases and controls for demographic characteristics and breast cancer risk factors by race, the Nashville Breast Health Study.

	Whites			Blacks		
	Cases (n = 1951)%	Controls (n = 1960)%	p-value	Cases (n = 660)%	Controls (n = 345)%	p-value
Age (yrs) (Mean±Standard deviation)	54.7	53.1	<0.0001	54.2	50.6	<0.0001
Education						
≤ High school	31.1	27.3	0.03	37.0	27.5	0.009
Some college	29.3	30.7		34.1	37.4	
≥ College	39.6	42.0		28.9	35.1	
Income						
≤ $20,000	8.8	6.0	0.0001	29.7	20.3	0.006
$20,001–40,000	16.4	14.2		26.8	28.7	
$40,001–60,000	20.9	23.7		17.8	18.8	
>$60,000	50.0	53.3		22.5	30.2	
Missing	3.9	2.8		3.2	2.0	
Breast cancer in first degree relatives	20.3	14.5	<0.0001	20.7	12.5	0.001
History of benign breast disease	48.5	34.3	<0.0001	31.9	24.1	0.01
Parous	84.0	84.4	0.74	85.4	84.1	0.56
Age at first live birth among parous (yrs)						
≤20	28.1	24.8	0.07	51.0	46.6	0.52
21–25	34.8	36.9		26.6	31.4	
26–30	23.5	25.9		15.1	14.8	
>30	13.6	12.4		7.3	7.2	
Age at menarche (years)						
≤11	21.5	20.5	0.005	26.7	29.1	0.48
12	27.3	27.0		28.1	30.2	
13	30.5	27.3		21.1	20.6	
≥14	20.7	25.2		24.1	20.1	
Alcohol intake	19.5	21.7	0.08	13.4	10.8	0.24
Cigarette smoking	42.2	42.2	0.98	39.6	35.1	0.17
Use of oral contraceptives	82.0	83.3	0.32	73.6	82.3	0.002
Use of hormone replacement therapy	55.7	52.3	0.04	37.5	37.1	0.91
Postmenopausal	65.5	63.2	0.14	67.4	58.4	0.005
Age at menopause among postmenopausal (yrs)						
<40	22.3	23.6	0.09	32.6	39.3	0.39
40–46	23.0	26.0		27.4	25.9	
47–50	26.6	22.6		19.9	16.4	
≥51	28.1	27.8		20.1	18.4	

## Results

Table 1 compares the demographic characteristics and breast cancer risk factors of white and black cases and controls. Among white women, cases were more likely than controls to be older and less educated, have lower income, a family history of breast cancer, a history of benign breast disease, a younger age at menarche, and have ever used hormone replacement therapy. Black cases tended to be older, less educated, have lower income, a family history of breast cancer, a history of benign breast disease, have never used oral contraceptives, and to be postmenopausal when compared to black controls. Black women appeared to be more likely than white women to experience menopause prior to age 40 years.

Table 2 presents the main effects of energy-related indicators on breast cancer risk by race and menopausal status adjusted for confounding. Among premenopausal white and black women, there was little effect of adult exercise or occupational activity on breast cancer risk. There was an increased odds of premenopausal breast cancer associated with increasing energy intake among black women (highest quartile relative to lowest quartile odds ratio [OR] 2.0, 95% confidence interval [CI] 1.0–3.8, p for trend = 0.05), but not among white women (OR 1.1, 95% CI 0.8–1.5, p for trend = 0.64) (p for interaction = 0.76). Relative to normal weight women, white women whose BMI was 25.0–29.9 kg/m^2^ (OR 1.4, 95% CI 1.1–1.8) and black women whose BMI was >30.0 kg/m^2^ (OR 2.2, 95% CI 1.1–4.6) were at increased risk of premenopausal breast cancer. For postmenopausal women, there was a similar reduction in odds of breast cancer associated with adult exercise and occupational activity among both white (highest tertile relative to none—exercise OR 0.8, 95% CI 0.6–1.0, p for trend = 0.05; occupational activity OR 0.6, 95% CI 0.4–0.9, p for trend = 0.10) and black (exercise OR 0.7, 95% CI 0.4–1.1, p for trend = 0.07; occupational activity OR 0.7, 95% CI 0.3–1.4, p for trend = 0.97) (exercise p for interaction = 0.29; occupational activity p for interaction = 0.30) women. Neither energy intake nor BMI was related to postmenopausal breast cancer. Weight change during adulthood was not related to pre- or postmenopausal breast cancer regardless of race.

**Table 2:** Odds ratios (ORs) and 95% confidence intervals for the associations of energy-related indicators with breast cancer by race and menopausal status, the Nashville Breast Health Study.

	Whites			Blacks		
	Premenopausal			Premenopausal		
	Cases (n = 672)	Controls (n = 720)	OR (95% CI) ^a^	Cases (n = 214)	Controls (n = 143)	OR (95% CI)^a^
Adult exercise (MET hrs/day)						
0	290	285	1.0 (referent)	108	68	1.0 (referent)
0.1–1.4	126	144	0.9 (0.7–1.2)	35	26	0.8 (0.5–1.5)
1.5–3.3	132	142	1.0 (0.7–1.3)	35	28	0.8 (0.4–1.5)
≥3.4	124	149	0.8 (0.6–1.1)	36	21	1.1 (0.6–2.0)
P for trend			0.26			0.90
P for interaction						0.16
Standing and walking at work (hrs/day)						
0	26	36	1.0 (referent)	10	7	1.0 (referent)
0.1–2.0	256	261	1.3 (0.7–2.2)	67	47	1.2 (0.4–3.6)
2.1–5.0	149	176	1.1 (0.6–1.9)	44	33	1.0 (0.3–3.0)
≥5.1	181	182	1.3 (0.8–2.3)	85	46	1.4 (0.5–4.2)
P for trend			0.58			0.46
P for interaction						0.73
Energy intake (kcal/d)						
<1187	146	161	1.0 (referent)	34	31	1.0 (referent)
1188–1548	147	167	1.0 (0.7–1.4)	31	24	1.5 (0.7–3.3)
1549–2035	154	174	1.0 (0.7–1.3)	27	17	2.1 (0.9–4.9)
≥2036	148	147	1.1 (0.8–1.5)	73	44	2.0 (1.0–3.8)
P for trend			0.64			0.05
P for interaction						0.76
BMI (kg/m^2^)						
<25.0	325	404	1.0 (referent)	37	28	1.0 (referent)
25.0–29.9	199	164	1.4 (1.1–1.8)	65	49	1.2 (0.6–2.3)
30.0–34.9	83	86	1.0 (0.7–1.4)	56	28	2.2 (1.1–4.6)
≥35	65	65	1.1 (0.7–1.6)	56	37	1.6 (0.8–3.2)
P for trend			0.87			0.17
P for interaction						0.91
Weight change since age 18 (lbs)^c^						
≤0	65	91	1.0 (referent)	14	9	1.0 (referent)
1–22	215	231	1.3 (0.9–2.0)	27	23	0.7 (0.2–2.0)
23–45	205	208	1.3 (0.9–1.9)	52	46	0.6 (0.2–1.7)
≥46	186	189	1.2 (0.8–1.8)	119	62	1.0 (0.4–2.8)
P for trend			0.68			0.27
P for interaction						0.91
	Postmenopausal			Postmenopausal		
	Cases (n = 1273)	Controls (n = 1237)	OR (95% CI)^b^	Cases (n = 442)	Controls (n = 201)	OR (95% CI)^b^
Adult exercise (MET hrs/day)						
0	619	548	1.0 (referent	267	101	1.0 (referent)
0.1–1.4	200	228	0.8 (0.6–1.0)	66	37	0.7 (0.5–1.2)
1.5–3.6	262	237	1.0 (0.8–1.2)	56	31	0.7 (0.4–1.2)
≥3.7	192	224	0.8 (0.6–1.0)	53	32	0.7 (0.4–1.1)
P for trend			0.05			0.07
P for interaction						0.29
Standing and walking at work (hrs/day)						
0	92	59	1.0 (referent)	47	12	1.0 (referent)
0.1–2.0	345	374	0.7 (0.5–0.9)	76	51	0.5 (0.2–1.0)
2.1–5.0	251	236	0.8 (0.5–1.1)	87	58	0.4 (0.2–0.9)
≥5.1	264	299	0.6 (0.4–0.9)	138	54	0.7 (0.3–1.4)
P for trend			0.10			0.97
P for interaction						0.30
Energy intake (kcal/d)						
<1090	302	279	1.0 (referent)	99	48	1.0 (referent)
1091–1430	278	294	0.9 (0.7–1.1)	68	33	1.0 (0.6–1.8)
1431–1916	320	298	1.0 (0.8–1.3)	73	29	1.3 (0.7–2.3)
≥1917	284	255	1.1 (0.8–1.3)	131	72	0.9 (0.6–1.5)
P for trend			0.40			0.85
P for interaction						0.61
BMI (kg/m^2^)						
<25.0	493	496	1.0 (referent)	75	37	1.0 (referent)
25.0–29.9	433	389	1.1 (0.9–1.3)	129	61	1.0 (0.6–1.7)
30.0–34.9	223	201	1.1 (0.9–1.4)	123	49	1.2 (0.7–2.0)
≥35	121	150	0.8 (0.6–1.1)	113	53	1.0 (0.6–1.7)
P for trend			0.67			0.90
P for interaction						0.43
Weight change since age 18 (lbs)^c^						
≤0	71	93	1.0 (referent)	23	10	1.0 (referent)
1–31	406	405	1.2 (0.8–1.6)	79	41	0.8 (0.3–2.1)
32–60	460	396	1.3 (0.9–1.9)	138	60	0.9 (0.4–2.3)
≥61	329	342	1.1 (0.8–1.6)	200	88	0.9 (0.4–2.2)
P for trend			0.76			0.90
P for interaction						0.62

^a^Odds ratio (OR) and 95% confidence interval (CI) adjusted for age, education, history of breast cancer in first degree relatives, OC use, and age at menarche.

^b^Additionally adjusted for HRT use.

^c^Additionally adjusted for weight at age 18.

When we analyzed these associations by tumor ER status, the increased premenopausal breast cancer risk associated with energy intake in black women was more pronounced for ER negative than for ER positive cancer. In contrast, the reduction in risk of postmenopausal breast cancer associated with adult exercise and occupational activity in white and black women was more pronounced, although not significantly, among women with ER positive than ER negative cancer (exercise p for interaction = 0.10; occupational activity p for interaction = 0.30) (S1 Table).

Table 3 presents the joint effects of other energy-related indicators (energy intake, BMI and weight change) on the adult exercise and breast cancer association by race and menopausal status adjusted for confounding. There was little effect of energy intake or BMI on the adult exercise and breast cancer association, regardless of menopausal status. Weight change modified the effect of adult exercise on premenopausal breast cancer among black women (p for interaction = 0.03), but not among white women. Black women in the highest category of adult exercise and weight change (OR 3.5, 95% CI 1.3–9.4) and in the lowest category of adult exercise and weight change (OR 3.6, 95% CI 1.2–11.2) were at increased risk of premenopausal breast cancer; however, the wide confidence intervals argue for cautious interpretation. White women who did not exercise and gained 32 to 60 pounds were at increased risk of postmenopausal breast cancer (OR 1.4, 95% CI 1.1–1.8). Although not significant, black women who did not exercise were at similarly increased risk (OR 1.5, 95% CI 0.7–3.4) of postmenopausal breast cancer across all categories of weight change.

**Table 3:** Odds ratios (ORs) and 95% confidence intervals for the joint associations of energy-related indicators with breast cancer by race and menopausal status, the Nashville Breast Health Study.

	Whites			Blacks		
	Premenopausal^a^			Premenopausal^a^		
	Adult exercise (MET hrs/day)			Adult exercise (MET hrs/day)		
	≥1.5	0.1–1.4	0	≥1.4	0.1–1.4	0
Energy intake (kcal/d)						
<1548	1.0 (referent)	1.0 (0.6–1.5)	1.2 (0.8–1.7)	1.0 (referent)	0.9 (0.3–2.6)	0.9 (0.4–2.2)
	111/137	56.70	126/121	21/17	13/11	31/27
1548–2035	0.9 (0.6–1.4)	1.3 (0.7–2.1)	1.1 (0.7–1.7)	0.9 (0.3–2.8)	1.8 (0.3–12.3)	3.0 (0.9–10.1)
	58/74	35/35	61/65	8/10	4/2	15/5
≥2036	1.2 (0.7–1.8)	1.1 (0.6–2.1)	1.3 (0.8–1.9)	1.9 (0.8–5.0)	0.9 (0.3–2.9)	1.5 (0.6–3.5)
	56/57	26/27	66/63	29/14	9/9	35/21
P for interaction			0.88			0.30
BMI (kg/m^2^)						
<30.0	1.0 (referent)	1.2 (0.8–1.6)	1.2 (0.9–1.5)	1.0 (referent)	0.8 (0.3–1.8)	1.2 (0.6–2.3)
	227/263	94/100	203/205	38/29	16/17	48/31
30.0–34.9	1.1 (0.6–2.1)	0.8 (0.4–1.5)	1.1 (0.7–1.7)	1.7 (0.6–4.8)	2.3 (0.7–7.5)	2.1 (0.9–4.9)
	22/21	20/26	41/39	14/8	12/5	30/15
≥35	1.1 (0.4–3.3)	0.8 (0.3–1.6)	1.1 (0.7–1.8)	1.8 (0.7–4.7)	1.3 (0.3–5.2)	1.3 (0.6–2.7)
	7/7	12/17	46/41	19/11	7/4	30/22
P for interaction			0.74			0.27
Weight change since age 18 (lbs)^c^						
<23	1.0 (referent)	1.3 (0.8–2.0)	1.3 (0.9–1.8)	1.0 (referent)	2.7 (0.7–11.2)	3.6 (1.2–11.2)
	130/164	44/47	106/111	13/17	7/5	21/10
23–45	1.1 (0.7–1.6)	1.6 (1.0–2.6)	1.0 (0.7–1.5)	2.1 (0.7–5.9)	0.8 (0.2–2.8)	1.7 (0.7–4.6)
	79/81	51/40	75/87	19/15	6/10	27/21
≥46	1.2 (0.7–1.9)	0.6 (0.4–1.1)	1.4 (0.9–2.0)	3.5 (1.3–9.4)	2.6 (0.9–7.5)	2.3 (0.9–5.6)
	46/46	31/56	109/87	38/15	22/11	59/36
P for interaction			0.91			**0.03**
	Postmenopausal^b^			Postmenopausal^b^		
	Adult exercise (MET hrs/day)			Adult exercise (MET hrs/day)		
	≥1.5	0.1–1.4	0	≥1.5	0.1–1.4	0
Energy intake (kcal/d)						
<1430	1.0 (referent)	0.8 (0.6–1.2)	1.1 (0.9–1.5)	1.0 (referent)	1.2 (0.5–2.8)	1.3 (0.7–2.4)
	217/219	89/110	274/244	46/28	27/13	94/40
1430–1916	0.9 (0.6–1.2)	1.2 (0.8–1.9)	1.4 (1.0–1.8)	2.2 (0.8–6.4)	1.0 (0.3–3.4)	1.5 (0.7–3.1)
	105/126	65/56	150/116	20/6	7/5	46/18
≥1917	1.3 (0.9–1.8)	0.7 (0.5–1.2)	1.3 (0.9–1.7)	0.7 (0.3–1.5)	1.0 (0.4–2.3)	1.4 (0.7–2.6)
	98/81	35/50	151/124	29/24	22/14	80/34
P for interaction			0.71			0.33
BMI (kg/m^2^)						
<30.0	1.0 (referent)	0.9 (0.7–1.2)	1.2 (1.0–1.4)	1.0 (referent)	0.9 (0.4–1.9)	1.4 (0.8–2.5)
	385/387	145/159	396/339	62/36	27/19	115/43
30.0–34.9	1.0 (0.7–1.5)	0.9 (0.5–1.5)	1.2 (0.9–1.7)	1.2 (0.6–2.7)	1.7 (0.6–4.8)	1.4 (0.8–2.6)
	53/52	31/36	139/113	29/13	17/6	77/30
≥35	0.7 (0.4–1.4)	0.8 (0.5–1.3)	0.9 (0.6–1.2)	0.8 (0.3–1.8)	1.0 (0.4–2.3)	1.5 (0.8–2.7)
	16/22	24/33	81/95	18/13	22/12	73/28
P for interaction			0.73			0.99
Weight change since age 18 (lbs)^c^						
<32	1.0 (referent)	1.1 (0.8–1.6)	1.1 (0.9–1.5)	1.0 (referent)	0.9 (0.3–2.6)	1.5 (0.7–3.4)
	223/245	78/82	176/171	28/18	13/10	61/23
32–60	1.2 (0.9–1.6)	0.9 (0.6–1.3)	1.4 (1.1–1.8)	1.2 (0.5–2.7)	1.1 (0.4–3.0)	1.5 (0.7–3.3)
	153/135	73/83	234/178	41/21	21/11	76/28
≥61	1.0 (0.7–1.4)	0.9 (0.6–1.3)	1.1 (0.9–1.5)	1.0 (0.4–2.2)	1.2 (0.5–2.9)	1.5 (0.7–2.9)
	76/81	49/63	204/198	40/22	32/16	128/50
P for interaction			0.95			0.90

^a^Odds ratio (OR) and 95% confidence interval (CI) adjusted for age, education, history of breast cancer in first degree relatives, OC use, and age at menarche.

^b^Odds ratio (OR) and 95% confidence interval (CI) additionally adjusted for HRT use.

^c^Additionally adjusted for weight at age 18.

## Discussion

Our finding of a reduced risk of breast cancer associated with adult exercise in the past 10 years among postmenopausal women, but not among premenopausal women, is in agreement with previous studies conducted among white women [5–6, 8–9]. In some studies of white [41–42], Asian [4], and black [25, 32, 35] women the reduction in risk was similar for women diagnosed pre- and postmenopausally. Bernstein et al. [33] reported comparable reductions in breast cancer risk associated with lifetime physical activity among white and black women, but did not stratify by menopausal status. Cohen et al. [7] matched on menopausal status and identified a reduced risk of breast cancer associated with total physical activity among white women, but not among black women. Ratnasinghe et al. [34] found stronger reductions in breast cancer risk among black than white women and among postmenopausal than premenopausal women. The reduction in postmenopausal breast cancer associated with occupational activity among white and black women seen in our study and that of John et al. [32] may be a function of the healthy worker effect, since women had to have been employed during the past 5 years. We saw a slightly greater reduction in postmenopausal breast cancer associated with physical activity among white and black women with ER positive than ER negative tumors which agrees with some [6–7, 33, 43], but not all studies [5, 34, 44] that investigated hormone receptor status. Possible explanations for differing findings across studies are the study design, the definition of physical activity, the time period investigated, and the evaluation of potential effect modifiers.

Overall there was a suggested increase in premenopausal breast cancer among black women associated with increased energy intake, which was significant for women with ER negative tumors, but not for women with ER positive tumors. To our knowledge, this is the first time this association has been examined jointly by race, menopausal status and hormone receptor status. This finding partially agrees with Zhang et al. [12] who reported an elevated breast cancer risk associated with total energy intake among premenopausal, but not postmenopausal, women. However, Zhang et al. [12] found this for women with ER positive rather than ER negative tumors. Silvera et al. [10] reported a significant positive trend in energy intake and premenopausal, but not postmenopausal, breast cancer in a study of Canadian women that did not stratify by race. Shin et al. [4] found no association between energy intake and breast cancer in their study of Chinese women. In their study of postmenopausal women who participated in the Prostate, Lung, Colorectal, and Ovarian Cancer Screening Trial, Sue et al. [11] reported a significant positive trend in energy intake and breast cancer among women at study entry, but not five years after study entry. Energy intake in the present study and others is typically based on a self-administered food frequency questionnaire which may result in underreporting, thereby adding to the inconclusive nature of results for the energy intake and breast cancer association [4, 10–11].

Contrary to most previous studies [13, 15–16, 18–21, 29–30], we found an elevation in premenopausal breast cancer associated with overweight in white women and with obesity in black women but no effect of BMI on postmenopausal breast cancer in women of either race. The results of studies of BMI and breast cancer conducted among black women tend to differ from those conducted among white women. Mayberry et al. [24] reported an elevated risk of breast cancer associated with high BMI among women diagnosed between ages 20 and 30, but not among women diagnosed at ages 40 to 54. Adams-Campbell et al. [25] found reductions in risk of pre- and postmenopausal breast cancer associated with high BMI. In a case-control study conducted among black women diagnosed between 1995 and 1998 in three large metropolitan counties of Tennessee, Zhu et al. [27] reported non-significant and significant elevations in risk of pre- and postmenopausal breast cancer, respectively, associated with high BMI. Palmer et al. [28] found a reduction in risk of premenopausal breast cancer, but no relation for postmenopausal breast cancer associated with high BMI. A recent meta-analysis of high BMI conducted among premenopausal women identified a reduced risk for breast cancer among Caucasian and African women, but an elevated risk among Asian women [18]. Although we did not find any differences in risk by hormone receptor status, another meta-analysis of high BMI reported a reduced risk for premenopausal breast cancer and an increased risk for postmenopausal breast cancer that was limited to women with ER positive tumors [45]. Our collection of information on height and weight was based on self-report over the telephone and reflects the trend toward increasing weight in white and black women which required a separate category for severe obesity. The most likely explanation for these elevated risks is misclassification since there was no association in white women who were obese or severely obese, and while the elevated risk in black women was present for severe obesity, it was no longer significant.

We did not see an association between adult weight change and pre- or postmenopausal breast cancer. Adult weight gain has been linked to an increased risk of postmenopausal breast cancer [4, 22–23, 46–47], but no association has been found for premenopausal breast cancer [22, 48]. The previous study of black women conducted in Tennessee reported non-significant elevations in risk of pre- and postmenopausal breast cancer associated with adult weight gain [27]. In agreement with a study of premenopausal women [48] and postmenopusal women [47], we found no difference by hormone receptor status. Similar to current BMI, self-report of weight at age 18 which was used to calculate adult weight change, is prone to misclassification.

Unlike previous studies, we did not identify any differences in breast cancer risk associated with physical activity by BMI [1–2, 5], or among women who gained [4] or lost [5] weight during adulthood.

Limitations of our study included self-report of physical activity, energy intake, current weight and height, and recall of weight at age 18 which are prone to misclassification. In addition, we were unable to collect other energy-related indicators such as waist-hip ratio and percent body fat, and our definition of weight change was based on a very wide range. Nor did we collect data on activities of daily living and housework which would have allowed us to sum physical activity for a more complete definition of total physical activity [4]. Our failure to collect information on physical activity for more than 10 years prior precluded our ability to examine the recognized effect of early adulthood physical activity on breast cancer risk [33]. However, we saw a stronger association for occupational activity which pertained to the past 5 years than for adult exercise which pertained to the past 10 years and was more likely to have included the premenopausal period. The fairly low response rates among cases and controls somewhat limited the generalizability of results. The joint effects analysis among black women was based on very small numbers. Lastly, our many subgroup analyses may have led to chance statistically significant findings.

Our study is one of the largest to investigate the joint effect of energy-related indicators on premenopausal and postmenopausal breast cancer among white and black women, separately. We adjusted for known breast cancer risk factors and stratified by race and menopausal status.

Our results indicate the effect of adult exercise on postmenopausal breast cancer risk is comparable in white and black women. White and black women should be encouraged to engage in more physical activity to reduce their risk of postmenopausal breast cancer.

## Supporting Information

S1 Table Odds ratios (ORs) and 95% confidence intervals for the associations of energy-related indicators with breast cancer by tumor estrogen receptor status, race and menopausal status, the Nashville Breast Health Study. (DOCX)
