# Supplementary material for: Energy-Related Indicators and Breast Cancer Risk among White and Black Women
Source: PLoS One. 2015 Apr 30;10(4):e0125058. doi: 10.1371/journal.pone.0125058 (PMC4416034; doi:10.1371/journal.pone.0125058)
Supplement: S1 Table — (DOCX) [file pone.0125058.s001.docx]

| Table S1. Odds ratios (ORs) and 95% confidence intervals for the associations of energy-related indicators with breast cancer by tumor estrogen receptor status, race and menopausal status, the Nashville Breast Health Study | | | | | | | | | | | |
| --- | --- | --- | --- | --- | --- | --- | --- | --- | --- | --- | --- |
|  | | Whites | | | | | Blacks | | | | |
|  | | Premenopausal | | | | | Premenopausal | | | | |
|  | Controls (n=720) | | ER+ cases  (n=382) | OR (95% CI) ^a^ | ER- cases (n=125) | OR (95% CI) ^a^ | Controls (n=143) | ER+ cases (n=78) | OR (95% CI)^a^ | ER- cases (n=42) | OR (95% CI)^a^ |
| Adult exercise (MET hrs/day) |  | |  |  |  |  |  |  |  |  |  |
| 0 | 285 | | 167 | 1.0 (referent) | 53 | 1.0 (referent) | 68 | 38 | 1.0 (referent) | 26 | 1.0 (referent) |
| 0.1-2.2 | 220 | | 111 | 0.9 (0.7-1.2) | 45 | 1.2 (0.7-1.8) | 40 | 20 | 0.9 (0.5-1.8) | 9 | 0.6 (0.2-1.4) |
| ≥2.3 | 215 | | 104 | 0.8 (0.6-1.1) | 27 | 0.7 (0.4-1.2) | 35 | 20 | 1.2 (0.6-2.3) | 7 | 0.6 (0.2-1.5) |
| P for trend |  | |  | 0.24 |  | 0.25 |  |  | 0.76 |  | 0.18 |
| P for interaction |  | |  |  |  |  |  |  |  |  | 0.31 |
|  |  | |  |  |  |  |  |  |  |  |  |
| Standing and walking at work (hrs/day) |  | |  |  |  |  |  |  |  |  |  |
| 0 | 36 | | 21 | 1.0 (referent) | 4 | 1.0 (referent) | 7 | 1 | 1.0 (referent) | 6 | 1.0 (referent) |
| 0.1-3.0 | 321 | | 179 | 0.9 (0.5-1.6) | 58 | 1.6 (0.5-4.6) | 63 | 27 | 3.7 (0.4-33.6) | 12 | 0.3 (0.1-1.0) |
| ≥3.1 | 298 | | 148 | 0.8 (0.5-1.5) | 49 | 1.4 (0.5-4.2) | 63 | 45 | 5.8 (0.6-52.1) | 24 | 0.4 (0.1-1.6) |
| P for trend |  | |  | 0.47 |  | 0.96 |  |  | 0.06 |  | 0.92 |
| P for interaction |  | |  |  |  |  |  |  |  |  | 0.29 |
|  |  | |  |  |  |  |  |  |  |  |  |
| Energy intake (kcal/d) |  | |  |  |  |  |  |  |  |  |  |
| <1187 | 214 | | 118 | 1.0 (referent) | 38 | 1.0 (referent) | 39 | 21 | 1.0 (referent) | 8 | 1.0 (referent) |
| 1187-1744 | 233 | | 124 | 1.0 (0.7-1.3) | 42 | 1.0 (0.6-1.7) | 24 | 16 | 1.9 (0.8-4.7) | 5 | 1.2 (0.3-4.3) |
| ≥1745 | 202 | | 123 | 1.1 (0.8-1.5) | 35 | 1.0 (0.6-1.6) | 53 | 39 | 1.7 (0.9-3.6) | 27 | **2.8 (1.1-7.2)** |
| P for trend |  | |  | 0.67 |  | 0.90 |  |  | 0.16 |  | **0.02** |
| P for interaction |  | |  |  |  |  |  |  |  |  | 0.95 |
|  |  | |  |  |  |  |  |  |  |  |  |
| BMI (kg/m^2^) |  | |  |  |  |  |  |  |  |  |  |
| <25.0 | 404 | | 183 | 1.0 (referent) | 57 | 1.0 (referent) | 28 | 17 | 1.0 (referent) | 7 | 1.0 (referent) |
| 25.0-29.9 | 164 | | 111 | 1.3 (1.0-1.8) | 37 | 1.6 (1.0-2.4) | 49 | 18 | 0.7 (0.3-1.7) | 15 | 1.5 (0.5-4.2) |
| >30.0 | 151 | | 88 | 1.1 (0.8-1.5) | 31 | 1.4 (0.8-2.2) | 65 | 43 | 1.5 (0.7-3.3) | 20 | 1.8 (0.7-5.2) |
| P for trend |  | |  | 0.74 |  | 0.20 |  |  | 0.26 |  | 0.42 |
| P for interaction |  | |  |  |  |  |  |  |  |  | 0.61 |
|  |  | |  |  |  |  |  |  |  |  |  |
| Weight change since age 18 (lbs)^c^ |  | |  |  |  |  |  |  |  |  |  |
| ≤0 | 91 | | 36 | 1.0 (referent) | 8 | 1.0 (referent) | 9 | 7 | 1.0 (referent) | 3 | 1.0 (referent) |
| 0.1-34 | 339 | | 176 | 1.3 (0.8-2.0) | 69 | 2.3 (1.0-5.0) | 42 | 18 | 0.4 (0.1-1.4) | 8 | 0.5 (0.1-2.4) |
| ≥35 | 289 | | 169 | 1.3 (0.9-2.1) | 48 | 1.8 (0.8-4.0) | 89 | 53 | 0.6 (0.2-1.8) | 31 | 0.9 (0.2-4.0) |
| P for trend |  | |  | 0.30 |  | 0.60 |  |  | 0.93 |  | 0.43 |
| P for interaction |  | |  |  |  |  |  |  |  |  | 0.70 |
|  | | Postmenopausal | | | | | Postmenopausal | | | | |
|  | Controls (n=1237) | | ER+ cases (n=736) | OR (95% CI) ^a^ | ER- cases (n=232) | OR (95% CI) ^a^ | Controls (n=201) | ER+ cases (n=181) | OR (95% CI)^a^ | ER- cases (n=83) | OR (95% CI)^a^ |
| Adult exercise (MET hrs/day) |  | |  |  |  |  |  |  |  |  |  |
| 0 | 548 | | 345 | 1.0 (referent) | 114 | 1.0 (referent) | 101 | 113 | 1.0 (referent) | 46 | 1.0 (referent) |
| 0.1-2.3 | 353 | | 195 | 0.9 (0.7-1.1) | 65 | 0.9 (0.7-1.3) | 58 | 40 | 0.7 (0.4-1.1) | 18 | 0.8 (0.4-1.5) |
| ≥2.4 | 336 | | 196 | 0.9 (0.7-1.1) | 53 | 0.8 (0.6-1.2) | 42 | 28 | 0.6 (0.4-1.2) | 19 | 1.1 (0.5-2.1) |
| P for trend |  | |  | 0.32 |  | 0.24 |  |  | 0.07 |  | 0.95 |
| P for interaction |  | |  |  |  |  |  |  |  |  | 0.10 |
|  |  | |  |  |  |  |  |  |  |  |  |
| Standing and walking at work (hrs/day) |  | |  |  |  |  |  |  |  |  |  |
| 0 | 59 | | 64 | 1.0 (referent) | 11 | 1.0 (referent) | 12 | 21 | 1.0 (referent) | 6 | 1.0 (referent) |
| 0.1-4.0 | 534 | | 289 | 0.6 (0.4-0.8) | 91 | 0.9 (0.5-1.8) | 97 | 42 | 0.3 (0.1-0.7) | 28 | 0.5 (0.2-1.5) |
| ≥4.1 | 375 | | 184 | 0.5 (0.3-0.7) | 73 | 1.0 (0.5-2.0) | 66 | 69 | 0.7 (0.3-1.6) | 39 | 0.9 (0.3-2.7) |
| P for trend |  | |  | 0.01 |  | 0.77 |  |  | 0.38 |  | 0.34 |
| P for interaction |  | |  |  |  |  |  |  |  |  | 0.30 |
|  |  | |  |  |  |  |  |  |  |  |  |
| Energy intake (kcal/d) |  | |  |  |  |  |  |  |  |  |  |
| <1090 | 380 | | 237 | 1.0 (referent) | 79 | 1.0 (referent) | 57 | 54 | 1.0 (referent) | 28 | 1.0 (referent) |
| 1090-1634 | 394 | | 243 | 1.0 (0.8-1.2) | 72 | 0.9 (0.6-1.3) | 41 | 37 | 1.1 (0.6-2.0) | 15 | 0.8 (0.4-1.6) |
| ≥1635 | 352 | | 234 | 1.1 (0.9-1.4) | 74 | 1.0 (0.7-1.5) | 84 | 76 | 1.2 (0.7-2.0) | 37 | 0.8 (0.5-1.6) |
| P for trend |  | |  | 0.43 |  | 0.89 |  |  | 0.50 |  | 0.61 |
| P for interaction |  | |  |  |  |  |  |  |  |  | 0.65 |
|  |  | |  |  |  |  |  |  |  |  |  |
| BMI (kg/m^2^) |  | |  |  |  |  |  |  |  |  |  |
| <25.0 | 496 | | 301 | 1.0 (referent) | 84 | 1.0 (referent) | 37 | 30 | 1.0 (referent) | 14 | 1.0 (referent) |
| 25.0-29.9 | 389 | | 237 | 1.0 (0.7-1.2) | 78 | 1.1 (0.8-1.6) | 61 | 55 | 1.0 (0.6-1.9) | 27 | 1.2 (0.5-2.5) |
| >30.0 | 351 | | 196 | 0.9 (0.7-1.2) | 69 | 1.1 (0.8-1.6) | 102 | 96 | 1.1 (0.6-2.0) | 42 | 1.0 (0.5-2.1) |
| P for trend |  | |  | 0.68 |  | 0.47 |  |  | 0.70 |  | 0.92 |
| P for interaction |  | |  |  |  |  |  |  |  |  | 0.27 |
|  |  | |  |  |  |  |  |  |  |  |  |
| Weight change since age 18 (lbs)^c^ |  | |  |  |  |  |  |  |  |  |  |
| ≤0 | 93 | | 47 | 1.0 (referent) | 11 | 1.0 (referent) | 10 | 9 | 1.0 (referent) | 2 | 1.0 (referent) |
| 0.1-44 | 601 | | 358 | 1.0 (0.7-1.5) | 112 | 1.3 (0.7-2.5) | 69 | 50 | 0.9 (0.3-2.6) | 35 | 1.9 (0.4-10.3) |
| ≥45 | 542 | | 329 | 1.0 (0.7-1.5) | 106 | 1.4 (0.7-2.6) | 120 | 121 | 1.1 (0.4-3.3) | 46 | 1.5 (0.3-7.9) |
| P for trend |  | |  | 0.80 |  | 0.49 |  |  | 0.39 |  | 0.62 |
| P for interaction |  | |  |  |  |  |  |  |  |  | 0.23 |

^a^Odds ratio (OR) and 95% confidence interval (CI) adjusted for age, education, history of breast cancer in first degree relatives, OC use, and age at menarche.

^b^Additionally adjusted for HRT use.

^c^Additionally adjusted for weight at age 18.
